# Targeting Rho Associated Kinases in Leukemia and Myeloproliferative Neoplasms

**DOI:** 10.18632/oncotarget.664

**Published:** 2012-09-21

**Authors:** Raghuveer Singh Mali, Reuben Kapur

**Affiliations:** Department of Pediatrics, Indiana University School of Medicine, Indianapolis; Department of Pediatrics, Indiana University School of Medicine, Indianapolis

Myeloproliferative neoplasms (MPNs) and acute myeloid leukemia (AML) are heterogeneous hematologic malignancies which share the common characteristics of myeloid cell overproduction and aberrant differentiation. With the exception of chronic myelogenous leukemia (CML), therapeutic intervention for these diseases is largely ineffective. There are increasing examples of mutations in tyrosine kinases (TKs) that contribute to MPN as well as AML. Key examples are the well characterized BCR-ABL transgene causing constitutively activated Abl tyrosine kinase function in CML. FLT3 internal tandem duplications (ITDs) resulting in ligand-independent autophosphorylation in AML and activating KIT mutations seen in AML and in over 90% cases of systemic mastocytosis (SM). Although hyper-activation of several signaling molecules downstream from these tyrosine kinases has been reported; little is known about the identity of signaling molecules that are shared downstream from these mutations. Recent studies suggest that activating TK mutants of KIT, FLT3 and BCR-ABL contribute to hematopoietic cell transformation to a large extent via the hyper-activation of Rho kinases[1]. Rho kinases have been shown to be hyperactive in several cancers, including breast cancer, colorectal cancer as well as prostate cancer[2, 3]. Importantly, in pre-clinical models, ROCK inhibitors have demonstrated significant efficacy in repressing aspects of tumorgenesis, in particular metastasis[4, 5]; however, their role in MPNs and AML is only now beginning to emerge[1, 6].

Two separate genes encode for two isoforms of Rho kinase or Rho-associated coiled coil-containing protein kinases (ROCK), ROCK1 and ROCK2. Rho kinases are protein serine/threonine kinases that share significant sequence homology at the protein level. Approximately 65% overall sequence homology and about 92% in their kinase domains[4, 7]. To elucidate the physiologic role of Rho kinases, small molecule inhibitors have been developed[4]. Fasudil (HA-1077), Y27632 and H-1152P or dimethylfasudil (diMF) are most commonly used Rho-kinase selective inhibitors and function in an ATP-competitive manner[4]. These drugs therefore equally inhibit the activity of ROCK1 and ROCK2. Fasudil is the only Rho kinase inhibitor to date that has been approved for use in humans. In humans, Fasudil has been used for the treatment of cardiovascular indications including hypertension, angina and stoke and appears to be relatively safe. Although no significant side effects have been reported in patients administered with Fasudil; it is unclear at this time if the beneficial effects of this drug in these patients are mediated via the suppression of ROCK1, ROCK2 or both. This is an important consideration, given that ROCK1 and ROCK2 are ubiquitously expressed and studies demonstrating differences in the function of these kinases are beginning to emerge[8].

Although several studies have implicated ROCK in regulating metastasis using small molecule inhibitors, two recent studies point to an essential role for diMF as a therapeutic drug for treating diverse groups of hematologic malignancies including some forms of MPN and AML[1, 6]. Mali et al recently demonstrated constitutive activation of ROCK in cells bearing oncogenic forms of KIT, FLT3 and BCR-ABL, which was dependent on the PI3K and Rho GTPase pathway[1]. They further demonstrated that genetic or pharmacologic inhibition of ROCK in oncogene bearing cells impaired the growth of leukemic blasts and significantly prolonged the life span of mice with MPN. Furthermore, treatment of leukemic cells with diMF resulted in rapid dephosphorylation of a known downstream substrate of ROCK, myosin light chain (MLC), resulting in apoptosis of leukemic blasts. Inhibition of MLC in vivo showed significantly prolonged life span of oncogene bearing leukemic mice. Consistent with the anti-leukemic effect of diMF on oncogene bearing cells; treatment of leukemic mice with Fasudil, also resulted in significant improvement in the life span of oncogene bearing leukemic mice[1].

In an independent study, Wen et al showed that diMF enhances polyploidization and apoptosis in acute megakaryocytic leukemia (AMKL) blasts[6]. In this study, authors showed that diMF induces apoptosis and polyploidization in AMLK cells by inhibiting the activation of Aurora kinase A (AURKA). These are important findings given the fact that leukemic blasts from AMKL patients hyperproliferate and fail to differentiate or undergo polyploidization. Furthermore, authors showed that diMF not only induces polyploidization but also induces apoptosis in AMLK blasts. Interestingly, Fasudil treatment did not induce polyploidization of AMLK cells[6]. Thus, although ROCK has been shown to be the primary target of diMF in most cell types including in oncogene bearing leukemic blasts; in AMLK cells, data suggests that its main target might be AURKA. While, diMF clearly represses the activation of AURKA and AURKA plays an essential role in inducing polyploidization in AMLKs; ROCK is likely to play a role in contributing to the polyploidization in AMLKs as bone marrow cells deficient in the expression of ROCK1 also demonstrate enhanced polyploidization[6]. Therefore, experiments involving knockdown of ROCK1 and/or ROCK2 in AMLK cells along with knockdown of AURKA should be able to address the relative contribution of these kinases in polyploidization of malignant megakaryocytes.

While ROCK inhibitors including Fasudil, Y27632 and diMF have been excellent tools to dissect the role of ROCK in leukemogenesis, further experiments utilizing mice deficient in the expression of ROCK1, ROCK2 and both ROCK1 and ROCK2 will be needed to more precisely delineate the role of ROCK in initiation and progression of leukemia. In addition, it will be interesting to determine if additional downstream substrates of ROCK such as LIMK and Ezrin also contribute to leukemogenesis. Small molecule inhibitors as well as mice deficient in the expression of these kinases have been described and will likely function as useful tools to dissect their respective role(s) in leukemogenesis in future studies[9, 10]. An additional important question that remains to be answered relates to the involvement of ROCK1 and ROCK2 in regulating the growth and survival as well as actin based functions in leukemia initiating cells as well as their role in regulating drug resistant mutations of BCR-ABL in CML and/or FLT3 in AML. Once a better understanding of the individual role of ROCK1 and ROCK2 in leukemogenesis is established, the development of isoform specific ROCK inhibitors may be necessary, in light of the fact that patients treated with Fasudil, which inhibits both ROCK1 and ROCK2, demonstrate considerable lowering of their blood pressure. Thus, if ROCKs are going to be targeted as anti-leukemic agents either as a monotherapy or in combination with existing drugs, a better characterization of the relative contribution of ROCK1 and ROCK2 in disease progression is essential.
